# Absence of manganese superoxide dismutase delays p53-induced tumor formation^☆^

**DOI:** 10.1016/j.redox.2014.01.001

**Published:** 2014-01-13

**Authors:** Adam J. Case, Frederick E. Domann

**Affiliations:** a Free Radical and Radiation Biology Program, Department of Radiation Oncology, Holden Comprehensive Cancer Center, The University of Iowa, Iowa City, IA 52242, USA; b Department of Cellular and Integrative Physiology, University of Nebraska Medical Center, Omaha, NE 68198, USA

**Keywords:** MnSOD, manganese superoxide dismutase, FoxO, Forkhead family of transcription factors, Anti-oxidant, Pro-oxidant, Cancer, Oxidative stress, Redox

## Abstract

**Background:**

Manganese superoxide dismutase (MnSOD) is a mitochondrial antioxidant enzyme that is down-regulated in a majority of cancers. Due to this observation, as well as MnSOD's potent antioxidant enzymatic activity, MnSOD has been suggested as a tumor suppressor for over 30 years. However, testing this postulate has proven difficult due to the early post-natal lethality of the MnSOD constitutive knock-out mouse. We have previously used a conditional tissue-specific MnSOD knock-out mouse to study the effects of MnSOD loss on the development of various cell types, but long-term cancer development studies have not been performed. We hypothesized the complete loss of MnSOD would significantly increase the rate of tumor formation in a tissue-specific manner.

**Results:**

Utilizing a hematopoietic stem cell specific Cre-recombinase mouse model, we created pan-hematopoietic cell MnSOD knock-out mice. Additionally, we combined this MnSOD knock-out with two well established models of lymphoma development: B-lymphocyte specific Myc over-expression and conditional pan-hematopoietic cell p53 knock-out. Mice were allowed to age unchallenged until illness or death had occurred. Contrary to our initial hypothesis, the loss of MnSOD alone was insufficient in causing an increase in tumor formation, but did cause significant life-shortening skin pathology in a strain-dependent manner. Moreover, the loss of MnSOD in conjunction with either Myc overexpression or p53 knock-out did not accelerate tumor formation, and in fact delayed lymphomagenesis in the p53 knock-out model.

**Conclusions:**

Our findings strongly suggest that MnSOD does not act as a classical tumor suppressor in hematological tissues. Additionally, the complete loss of MnSOD may actually protect from tumor development by the creation of an unfavorable redox environment for tumor progression. In summary, these results in combination with our previous work suggest that MnSOD needs to be tightly regulated for proper cellular homeostasis, and altering the activity in either direction may lead to cellular dysfunction, oncogenesis, or death.

## Highlights

• The free radical theory of cancer postulates that loss of MnSOD promotes cancer.
• We created mouse models of malignancy with and without conditional loss of MnSOD.
• We show that MnSOD loss delays the onset of p53-dependent tumor development.
• Our data suggest that inhibition of MnSOD in tumor cells may slow tumor progression.

## Graphical abstract

**Figure:**
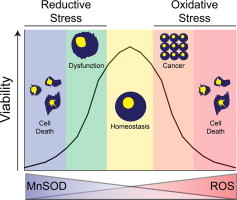

## Introduction

In 1979, Larry Oberley and Garry Buettner outlined the free radical theory of oncogenesis [1]. The theory stemmed from numerous observations that the superoxide dismutase class of enzymes, particularly the mitochondrial isoform manganese superoxide dismutase (MnSOD), was shown to be expressed at low levels in cancers of various cell types compared to their normal tissues of origin. The theory frameworks three predictions in regards to the role of MnSOD and cancer: (1) all cancers should possess decreased amounts of MnSOD activity compared to their normal tissue counterparts, (2) replacement of MnSOD in tumor cells should abrogate the malignant phenotype, and (3) normal cells that lose MnSOD will in turn become malignant. With very few exceptions, the first two postulates have overwhelming evidence demonstrating their validity in regards to MnSOD and cancer. In contrast, the third postulate defines MnSOD as a genuine tumor suppressor protein, and is the only postulate that remains unanswered to date.

The major limitation in addressing the third prediction has been a lack of a viable MnSOD knock-out model. Two separate constitutive MnSOD knock-out mice strains have been developed, and both demonstrate early post-natal lethality due to overt oxygen toxicity and system-wide organ failure [2], [3]. These observations have elucidated the critical function of MnSOD in mammalian development, but do not address the role of MnSOD in oncogenesis. To date, the most convincing evidence that MnSOD acts as a tumor suppressor protein is from MnSOD heterozygous mice. While these mice possess the same lifespan of wild-type mice, they have significantly more cancer (primarily lymphoma) at the end of their life [4]. While this evidence proposes a compelling argument for MnSOD as a tumor suppressor protein, the heterozygous mice still retain at least 50% MnSOD activity, and thus have not addressed the hypothesis that complete loss of MnSOD initiates tumor formation in a dose-dependent fashion and therefore acts as a classical tumor suppressor protein.

In 2002, Ikegami et al. created a conditional MnSOD knock-out mouse utilizing Cre/loxP technology [5]. Since this time, our group has been at the forefront of characterizing the effects of tissue-specific loss of MnSOD in an array of cell types [6], [7], [8], [9]. We have observed significant phenotypes due to the loss of MnSOD including severe immunodeficiency (T-lymphocytes), aberrant iron homeostasis (pan-hematopoietic cells), and epigenetic dysregulation (liver) in several models we have developed [7], [8], [9]. To our surprise, the complete loss of MnSOD in a tissue specific manner alone did not cause a significant increase in tumor formation in any of the animal models when aged [5], [6], [7], [8]. These data argue that MnSOD does not act as a tumor suppressing protein in regards to the initiation of oncogenesis, and is in direct contradiction with the third prediction of the free radical theory of cancer. Therefore, we hypothesized that MnSOD alone does not act as a classical tumor suppressor, but the loss of MnSOD may exacerbate the formation of cancer in an established model of oncogenesis. To address this hypothesis, we crossbred our mouse model of conditional pan-hematopoietic cell MnSOD knock-out with two respective mouse models of lymphoma development to understand if the loss of MnSOD accelerated the formation of cancer. We show for the first time that the loss of MnSOD either had no effect or prolonged lymphoma development in the respective models. Taken together, these data strongly suggest that the loss of MnSOD is likely not a casual event in the initiation of cancer, but instead may be down-regulated in fully initiated cancer cells to aid in tumor progression.

## Methods

Mice homozygous for the floxed MnSOD allele (*i.e.* B6.Cg-*Sod2*^*tm1*^, shorthand MnSOD^L/L^) were initially bred to mice expressing Cre-recombinase under the control of the vav promoter (*i.e.* B6.Cg-Tg-*Vav1-iCre*^*A2Kio*^/J, shorthand vav-Cre mice, Jackson Laboratories) to create hematopoietic stem cell specific knock-outs of MnSOD [7], [10]. These mice were sequentially bred to either an oncogenic model of splenic lymphoma (*i.e.* B6.Cg-Tg-*iMyc*^*Eμ*^, shorthand iMyc^+/−^) or a conditional tumor suppressor knock-out model of thymic and splenic lymphoma (*i.e*. FVB.129-*Trp53*^*tm1Brn*^, shorthand p53^L/L^, NCI-Frederick Mouse Repository) [11], [12]. In all experiments, littermate controls were used to limit the effects of genetic variation amongst strains. Upon weaning, mice were analyzed by tail DNA to confirm appropriate genotype. Mice were observed until death with no additional challenges. Upon death or illness, all mice were examined by full necropsy and cause of death recorded. Kaplan-Meier with Log-Rank analysis was performed and non-cancer deaths were appropriately censored. Causes of death were compared between groups and analyzed by unpaired two-tail Student's *t*-test. A *p* value of less than 0.01 was considered significant. All work was performed under the approval of the Institutional Animal Care and Use Committee at the University of Iowa.

## Results and discussion

**Fig. 1:**
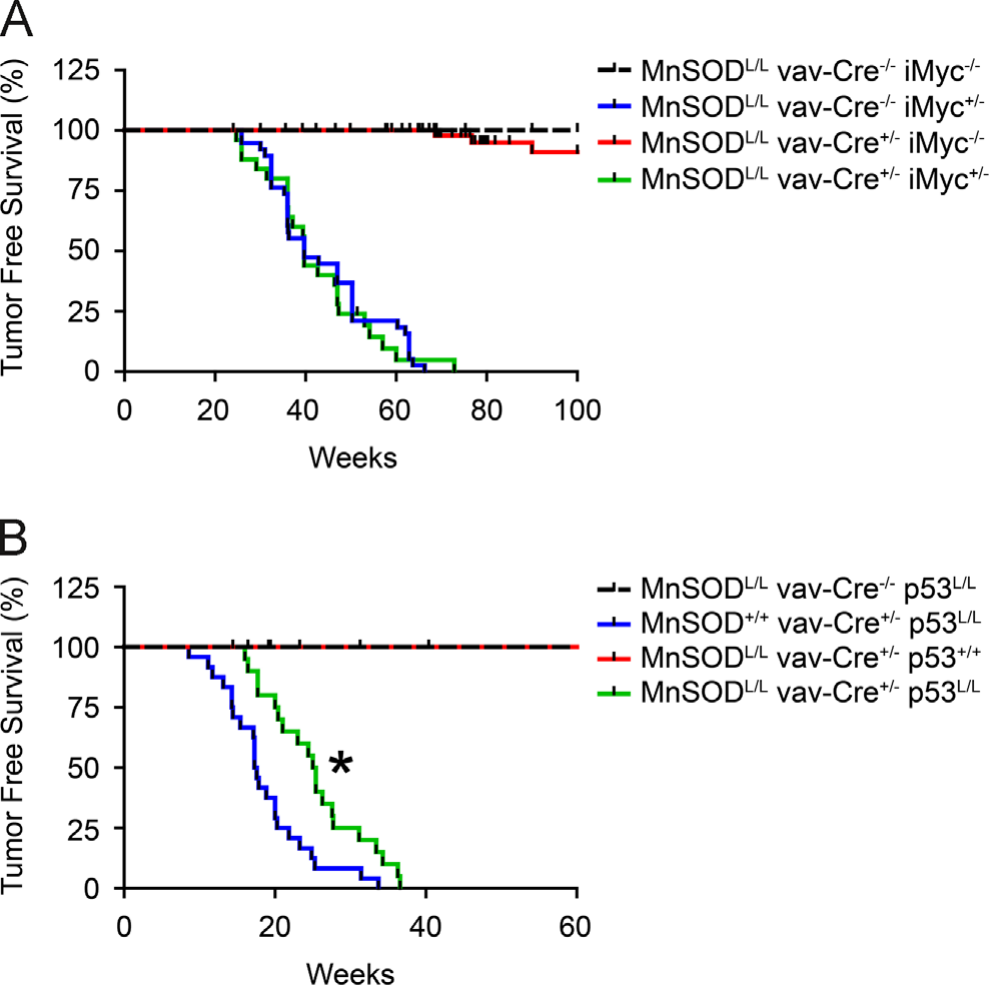
MnSOD loss prolongs tumor development in a p53 knock-out cancer model. (A) Conditional hematopoietic MnSOD knock-out mice (MnSOD^L/L^ vav-Cre^+/−^) were crossed with mice over-expressing Myc targeted specifically to B-lymphocytes (iMyc^+/−^). Survival studies demonstrate no significant changes in tumor formation with the loss of MnSOD in combination with Myc over-expression. (B) Conditional MnSOD knock-out mice (MnSOD^L/L^) were crossed with conditional p53 knock-out mice (p53^L/L^). Further breeding to the hematopoietic stem cell driven Cre-recombinase mouse (vav-Cre^+/−^) creates double conditional knock-outs for MnSOD and p53 targeted to hematopoietic tissues. Survival studies demonstrate the loss of MnSOD prolongs tumor approximately 10 weeks. Hash marks above lines indicate censored non-tumor deaths. ^⁎^*p*<0.01 by Log-Rank analysis.

We first examined the effects of MnSOD loss in an oncogenic model of splenic lymphoma. We have previously demonstrated that our hematological stem cell MnSOD knock-out is devoid of MnSOD and possesses increased mitochondrial oxidative stress in all hematological tissues such as bone marrow, spleen, and thymus [7]. By combining our hematological stem cell knock-out of MnSOD (MnSOD^L/L^ vav-Cre^+/−^) with a model of conditional B-lymphocyte Myc over-expression (iMyc^+/−^), we assessed the rate in which these animals developed splenic lymphoma. One-hundred percent of mice possessing MnSOD and the iMyc allele (MnSOD^L/L^ vav-Cre^−/−^ iMyc^+/−^) developed splenic lymphoma with a mean tumor free-survival time of approximately 45 weeks (Fig. 1A, Table 1). Surprisingly, the loss of MnSOD in conjunction with Myc over-expression (MnSOD^L/L^ vav-Cre^+/−^ iMyc^+/−^) did not significantly affect the rate of tumor formation (Fig. 1A, Table 1). These data suggest the loss of MnSOD does not act to promote tumor initiation in a Myc-driven model of B-cell lymphoma.

**Table 1:** Cause of death in aged mice containing MnSOD, iMyc, and p53 mutations.

	**MnSOD**^**L/L**^**vav-Cre**^**−/−**^**iMyc**^**−/−**^	**MnSOD**^**L/L**^**vav-Cre**^**−/−**^**iMyc**^**+/−**^	**MnSOD**^**L/L**^**vav-Cre**^**+/−**^**iMyc**^**−/−**^	**MnSOD**^**L/L**^**vav-Cre**^**+/−**^**iMyc**^**+/−**^
**Dermatitis**	0 (0.0%)	0 (0.0%)	**28 (47.5%)**⁎	0 (0.0%)
**Splenic lymphoma**	0 (0.0%)	**38 (100.0%)**⁎	3 (5.1%)	**25 (100.0%)**⁎
**Unknown**	7 (8.9%)	0 (0.0%)	8 (13.6%)	0 (0.0%)
**Termination of study**	72 (91.1%)	0 (0.0%)	20 (33.9%)	0 (0.0%)

**Total**	79	38	59	25

	**MnSOD**^**L/L**^**vav-Cre**^**−/−**^**p53**^**L/L**^	**MnSOD**^**+/+**^**vav-Cre**^**+/−**^**p53**^**L/L**^	**MnSOD**^**L/L**^**vav-Cre**^**+/−**^**p53**^**+/+**^	**MnSOD**^**L/L**^**vav-Cre**^**+/−**^**p53**^**L/L**^

**Dermatitis**	0 (0.0%)	0 (0.0%)	1 (1.8%)	0 (0.0%)
**Splenic lymphoma**	0 (0.0%)	**4 (16.7%)**⁎	1 (1.8%)	**7 (35.0%)**⁎
**Thymic lymphoma**	0 (0.0%)	**20 (83.3%)**⁎	0 (0.0%)	**13 (65.0%)**⁎
**Unknown**	5 (10.6%)	0 (0.0%)	4 (7.1%)	0 (0.0%)
**Termination of study**	42 (89.4%)	0 (0.0%)	50 (89.3%)	0 (0.0%)

**Total**	47	24	56	20

⁎ *p*<0.01 by Student's *t*-test compared to respective control animal (*e.g.* MnSOD^L/L^ vav-Cre^−/−^ iMyc^−/−^ or MnSOD^L/L^ vav-Cre^−/−^ p53^L/L^).

It was observed that the loss of MnSOD alone (MnSOD^L/L^ vav-Cre^+/−^ iMyc^−/−^) did not significantly increase the incidence of any type of hematological malignancy (Fig. 1A, Table 1). In contrast, while malignancy was not increased in these animals, significant life-shortening severe dermatitis was observed (Table 1). We have previously reported that the MnSOD^L/L^ vav-Cre^+/−^ suffer from stark anemia, porphyria, and potential immunodeficiency [7], [8]. At this time we cannot rule out any of these pathologies in the contribution to this skin pathology. Porphyria is a significant contributor to skin pathology, as porphyrin rings (macrocycles) are not properly metabolized and building up in dermal tissues [13]. In our MnSOD knock-out model, macrocycle build-up may be a major contributor to the skin pathology, but further studies are needed to confirm the presence of porphyrin rings in the dermal tissues. Additionally, C57BL/6 mice are prone to various forms of skin dermatitis, and the loss of MnSOD in the hematopoietic system may exacerbate this commonly observed endogenous phenotype. In 2007, Ronald DePinho's group reported increased life-shortening dermatitis in a C57BL/6 hematopoietic specific knock-out of all of the mammalian forkhead transcription factors (FoxOs) [14]. The FoxO family of transcription factors has been shown to control the expression of anti-oxidant enzymes including MnSOD, and as such the knock-outs displayed significant oxidative stress comparable to our MnSOD knock-out models. The results of our study extend these findings showing that hematological oxidative stress appears to have significant effects on proper skin maintenance, though mechanistically how this is achieved is still unclear.

Next, we examined the effect of MnSOD loss in a conditional hematological knock-out of the tumor suppressor gene p53 (p53^L/L^). We have previously reported that p53 activation was associated with the loss of MnSOD in T-lymphocytes, and contributed to accelerated apoptosis and a severe immunodeficient phenotype [8], therefore, we hypothesized a potential synergistic oncogenic effect with the loss of both p53 and MnSOD. Moreover, because both MnSOD and p53 conditional knock-out models are driven by Cre/loxP genetics, the combination allows for the loss of both genes in the same hematological cells as driven by the vav promoter. We first observed significant and rapid thymic and splenic lymphoma development in mice possessing only p53 knock-out (MnSOD^+/+^ vav-Cre^+/−^ p53^L/L^) with a mean tumor free-survival time of approximately 18 weeks (Fig. 1B, Table 1). Unexpectedly, the loss of both MnSOD and p53 (MnSOD^L/L^ vav-Cre^+/−^ p53^L/L^) significantly delayed tumor formation with a mean tumor free-survival of approximately 28 weeks (Fig. 1B, Table 1). In contrast to the previous model, the loss of MnSOD alone (MnSOD^L/L^ vav-Cre^+/−^ p53^+/+^) appeared to have no overt life-shortening pathologies like dermatitis, which suggests a potential strain-dependent phenotype. Overall, these data strongly imply that MnSOD does not act as a classical tumor suppressor protein even in conjunction with known tumor-driving mutations, and in fact the loss of this protein may be beneficial in the prevention of some types of cancers.

**Fig. 2:**
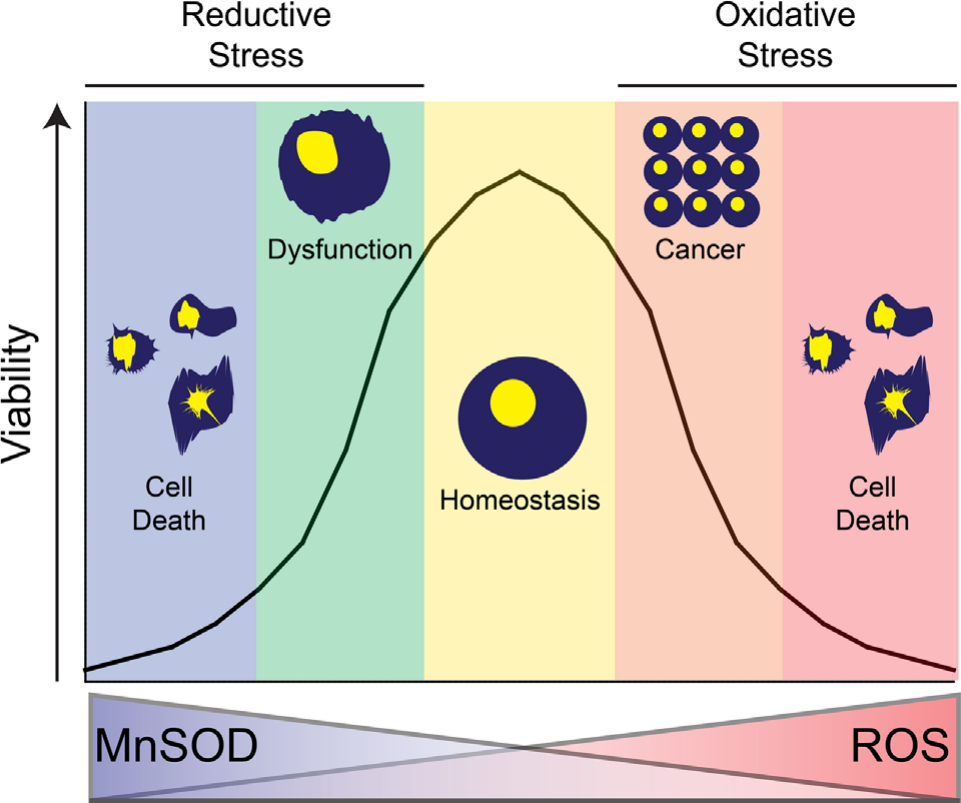
Redox balance is essential in the maintenance of cellular viability. In this study, we demonstrate that the complete loss of MnSOD prolongs the development of p53-induced cancer. This may be due to the fact that complete loss of MnSOD causes significant increases in oxidative stress, and thus causes cell death as opposed to cancer. In contrast, mice heterozygous for MnSOD marginally elevated levels of oxidative stress, and demonstrate an increased incidence of cancer. Conversely, excessive over-expression of MnSOD has demonstrated pathological and toxic effects in certain systems. Taken together, a fine balance is essential in cellular homeostasis, and disruption of the redox balance in either direction may hinder cellular viability and function.

Our findings presented here contradict the original free radical theory of cancer which predicts the loss of MnSOD would cause significant increases in tumor formation. Our data suggest that down-regulation of MnSOD prior to tumor formation leads to an unfavorable environment for cancer development, and may even protect from tumor development in certain models. One explanation for this is simply the overwhelming oxidative stress by the loss of MnSOD causes cells to die before they become malignant. Another possible reason for the loss of MnSOD which may delay tumor development is the increased susceptibility to the immune response. Once tumor cells are formed, they are often detected by the innate immune response, in which these cells attempt to destroy the malignant cells by an oxidative burst mechanism [15]. It could be postulated that the loss of MnSOD may increase the susceptibility to the innate immune response, and thus delay the development of systemic cancer. Taken together with our previous reports, these observations indicate the importance of a normal redox balance for proper cellular homeostasis, and that shifting this balance by altering MnSOD levels may lead to cellular death, dysfunction, and cancer on either end of the spectrum (Fig. 2, Graphical Abstract). Some groups have already attempted to exploit the redox balance by targeting cellular antioxidants to push cancers into an extreme pro-oxidant environment and cellular death [16], [17], [18]. While this strategy shows significant potential, the long term effects of inhibiting cellular antioxidants may have detrimental effects on homeostatic normal cells. While evidence of oxidative stress-induced hormesis has been described [19], the fine line between beneficial and pathological levels of pro-oxidants are currently not well defined.

## Conclusions and future work

In conclusion, we present the first observation that complete loss of MnSOD does not exacerbate tumor formation alone or in combination with two well established lymphoma-causing mutations, and moreover, may prevent tumorogenesis in some instances. Our results may be limited by the examination of the effects of MnSOD loss solely on the hematopoietic system. While these cells were shown to be the most susceptible to tumor formation in the MnSOD heterozygous mice [4], the complete loss of MnSOD has demonstrated negligible effects on tumor development. To address this limitation, we will create various conditional knock-outs of MnSOD in conjunction with tissue-specific tumor promoting mutations. We would hypothesize that each tissue will demonstrate different susceptibilities to cancer formation by the loss of MnSOD, and thus this information will prove critical in the potential targeting of specific cancers by antioxidant or pro-oxidant therapy in the future.

Another potential limitation of this study is the timing of the MnSOD knock-out. By using a hematopoietic stem cell promoter, the loss of MnSOD occurred prior to any tumor formation caused by either Myc over-expression or p53 loss. While our findings demonstrate interesting effects on tumor initiation in these models, these data fail to explain why a majority of tumor cell types down-regulate MnSOD over time. To address this, we are currently developing inducible models of MnSOD knock-out. In this manner, tumor formation can become initiated by a known cancer-causing mutation before temporally knocking-out MnSOD. These models allow for examining the effects of MnSOD loss on tumor progression, which is likely the stage of cancer that loss of MnSOD is a major contributor.

Finally, we observed an unexpected result that loss of MnSOD in the hematopoietic system led to increased dermatologic pathologies. This finding is highly intriguing, and the mechanism underlying the pathology is currently unknown. Numerous human skin pathologies have been linked to immune system dysfunction, but a connection to oxidative stress in the immune system has not been described. We will be performing comprehensive pathologic analyses on the skin lesions to understand their etiology, and potentially elucidate the link between oxidative stresses in the immune system to dermatologic maintenance. Overall, our data extend the understanding of the contribution of the redox environment in immune system function and lymphomagenesis, and challenge the dogma of MnSOD being causal in tumor development established more than three decades prior.

## Competing interests

The authors declare no competing financial interests.

## Authors' contributions

AJC and FED conceived experimental designs. AJC performed the research, and FED supervised the project. AJC and FED contributed equally in the preparation of the manuscript.

## References

[1] Oberley L.W., Buettner G.R. (1979). Role of superoxide dismutase in cancer: a review. Cancer Res..

[2] Lebovitz R.M., Zhang H., Vogel H., Cartwright J., Dionne L., Lu N., Huang S., Matzuk M.M. (1996). Neurodegeneration, myocardial injury, and perinatal death in mitochondrial superoxide dismutase-deficient mice. Proc. Natl. Acad. Sci. USA.

[3] Li Y., Huang T.T., Carlson E.J., Melov S., Ursell P.C., Olson J.L., Noble L.J., Yoshimura M.P., Berger C., Chan P.H. (1995). Dilated cardiomyopathy and neonatal lethality in mutant mice lacking manganese superoxide dismutase. Nat. Genet..

[4] Van Remmen H., Ikeno Y., Hamilton M., Pahlavani M., Wolf N., Thorpe S.R., Alderson N.L., Baynes J.W., Epstein C.J., Huang T.T. (2003). Life-long reduction in MnSOD activity results in increased DNA damage and higher incidence of cancer but does not accelerate aging. Physiol. Genomics.

[5] Ikegami T., Suzuki Y., Shimizu T., Isono K., Koseki H., Shirasawa T. (2002). Model mice for tissue-specific deletion of the manganese superoxide dismutase (MnSOD) gene. Biochem. Biophys. Res. Commun..

[6] Case A.J., Domann F.E. (2012). Manganese superoxide dismutase is dispensable for post-natal development and lactation in the murine mammary gland. Free Radic. Res..

[7] Case A.J., Madsen J.M., Motto D.G., Meyerholz D.K., Domann F.E. (2013). Manganese superoxide dismutase depletion in murine hematopoietic stem cells perturbs iron homeostasis, globin switching, and epigenetic control in erythrocyte precursor cells. Free Radic. Biol. Med..

[8] Case A.J., McGill J.L., Tygrett L.T., Shirasawa T., Spitz D.R., Waldschmidt T.J., Legge K.L., Domann F.E. (2011). Elevated mitochondrial superoxide disrupts normal T cell development, impairing adaptive immune responses to an influenza challenge. Free Radic. Biol. Med..

[9] Cyr A.R., Brown K.E., McCormick M.L., Coleman M.C., Case A.J., Watts G.S., Futscher B.W., Spitz D.R., Domann F.E. (2013). Maintenance of mitochondrial genomic integrity in the absence of manganese superoxide dismutase in mouse liver hepatocytes. Redox Biol..

[10] de Boer J., Williams A., Skavdis G., Harker N., Coles M., Tolaini M., Norton T., Williams K., Roderick K., Potocnik A.J., Kioussis D. (2003). Transgenic mice with hematopoietic and lymphoid specific expression of Cre. Eur. J. Immunol..

[11] Marino S., Vooijs M., van Der Gulden H., Jonkers J., Berns A. (2000). Induction of medulloblastomas in p53-null mutant mice by somatic inactivation of Rb in the external granular layer cells of the cerebellum. Genes Dev..

[12] Park S.S., Kim J.S., Tessarollo L., Owens J.D., Peng L., Han S.S., Tae Chung S., Torrey T.A., Cheung W.C., Polakiewicz R.D. (2005). Insertion of c-Myc into Igh induces B-cell and plasma-cell neoplasms in mice. Cancer Res..

[13] Dombeck T.A., Satonik R.C. (2005). The porphyrias. Emerg. Med. Clin. North Am..

[14] Tothova Z., Kollipara R., Huntly B.J., Lee B.H., Castrillon D.H., Cullen D.E., McDowell E.P., Lazo-Kallanian S., Williams I.R., Sears C. (2007). FoxOs are critical mediators of hematopoietic stem cell resistance to physiologic oxidative stress. Cell.

[15] Herberman R.B., Ortaldo J.R. (1981). Natural killer cells: their roles in defenses against disease. Science.

[16] Juarez J.C., Betancourt O., Pirie-Shepherd S.R., Guan X., Price M.L., Shaw D.E., Mazar A.P., Donate F. (2006). Copper binding by tetrathiomolybdate attenuates angiogenesis and tumor cell proliferation through the inhibition of superoxide dismutase 1. Clin. Cancer Res..

[17] Glasauer A., Sena L.A., Diebold L.P., Mazar A.P., Chandel N.S. (2014). Targeting SOD1 reduces experimental non-small-cell lung cancer. J. Clin. Invest..

[18] Welsh J.L., Wagner B.A., van't Erve T.J., Zehr P.S., Berg D.J., Halfdanarson T.R., Yee N.S., Bodeker K.L., Du J., Roberts L.J. (2013). Pharmacological ascorbate with gemcitabine for the control of metastatic and node-positive pancreatic cancer (PACMAN): results from a phase I clinical trial. Cancer Chemother. Pharmacol..

[19] Ristow M., Schmeisser S. (2011). Extending life span by increasing oxidative stress. Free Radic. Biol. Med..

